# Evaluation of Satisfaction With Care in Paediatric Intensive Care Units: Swedish Parents' Perspective

**DOI:** 10.1111/nicc.70086

**Published:** 2025-06-16

**Authors:** Karina Terp, Ulf Jakobsson, Janne Weis, Pia Lundqvist

**Affiliations:** ^1^ Department of Health Sciences Lund University Lund Sweden; ^2^ Center for Primary Health Care Research, Department of Clinical Sciences (Malmö) Lund University Lund Sweden; ^3^ Department of Neonatology Copenhagen University Hospital Rigshospitalet Copenhagen Denmark

**Keywords:** paediatric intensive care, parents, patient and family‐centred care, satisfaction

## Abstract

**Background:**

A child's hospitalization in a paediatric intensive care unit is a stressful experience for parents. Measuring parental satisfaction may indicate how parents are affected by the experience. Satisfaction with care is closely connected to the overall quality of care.

**Aim:**

The aim was to explore parental satisfaction with paediatric intensive care.

**Study Design and Method:**

A cross‐sectional study design was utilized at two PICUs in Sweden. Inclusion criteria were parents who spoke and understood Swedish and whose child was < 18 years old and hospitalized at the PICU for at least 48 h. Exclusion criteria were parents whose child died during the care period at the PICU. The questionnaire EMPATHIC‐30, grounded in the principles of family‐centred care, was the basis for the data collection. Descriptive statistics and a non‐parametric test (Mann–Whitney U‐test) were used to present the data.

**Results:**

A total of 234 questionnaires were distributed, and the response rate was 42.73%. Notably, 100 parents responded to the questionnaire, and 97 were included (mothers *n* = 50 and fathers *n* = 47). The parents' mean age was 36.26 years (SD 7.00) and ranged between 23 to 61 years. The results revealed high levels of parental satisfaction with the care provided according to total scale (5.53), domains (5.42–5.77), as well as single items (4.44–5.99). The EMPATHIC‐30 scores indicated that parents felt well‐informed about their child's condition and received emotional support from health care professionals. Additionally, the study identified areas for improvement, such as the need for enhanced communication, being actively involved in the process of decision‐making as well as being involved/prepared before discharge from the PICU.

**Conclusion:**

Parents expressed high satisfaction across the five domains and for the total scale. However, areas for improvement were identified for individual items. Factors that tended to give lower satisfaction were communication between health care professionals and parents, as well as parents' active involvement in decision‐making regarding their child's care.

**Relevance to Clinical Practice:**

The results of this study emphasize the need for health care organizations to prioritize communication strategies for parents of children cared for at PICUs. By applying a person‐centred two‐way communication, health care professionals can facilitate open and transparent communication with parents, which can promote parental involvement in care and decision‐making.

**Summary**

- What is known about this topic
  ○ Parents of children hospitalized in paediatric intensive care units can experience acute as well as post‐traumatic stress.
  ○ Parents with higher levels of care satisfaction experience less stress.
  ○ One way to improve parental satisfaction with care is by working according to the principles of patient and family‐centred care.
- What this paper adds
  ○ It provides knowledge about parents' satisfaction with care when their child has been hospitalized in Swedish PICUs.
  ○ It highlights the critical areas health care professionals should address to enhance parent satisfaction, such as clear communication and information about medications, and to ensure opportunities for closeness during intensive procedures, in alignment with family‐centred care principles.
  ○ It illustrates that parents' challenges in the PICU could be encountered through timely person‐centred communication, especially during high‐stress care moments such as intensive care procedures and care transitions.

## 1 Introduction

Children undergoing intensive care are in a vulnerable situation and their parents' presence is vital as they become the child's secure base. When a child requires intensive care, it also means that the parents experience a vulnerable situation; hence they need support and help to deal with their anxiety and have their needs met [1, 2]. It has been found that parents of children in need of intensive care request a professional attitude from health care professionals, as well as the opportunity to be involved in and informed about their child's care [3]. According to Swedish regulations and the United Nations Convention on the Rights of the Child, guardians have the right to participate in and influence their child's care [4, 5]. Measuring satisfaction with care is one way to ensure that the treatment provided meets parents' as well as the child's needs. Satisfaction with care is closely connected to the overall patient experience. It encompasses various aspects of the health care journey, including interactions with health care providers, the quality of medical treatment, the physical environment of health care facilities, and the communication and information provided to the patient [6]. Perceived satisfaction with care can also be seen as an indicator of quality of care [7, 8, 9]. Predictors affecting satisfaction with care are, among other things, the child's severity of illness, length of hospitalization, and collaboration between family members and health care professionals [10]. Other factors that may influence parents' satisfaction with care are the child's and parent's age, the parent's level of education, the number of children in the family, and previous health care experience [11].

Previous research indicates that parents and families of children cared for in a paediatric intensive care unit (PICU) experience the situation as stressful [12, 13]. Hospitalization has an impact on the family's daily life both during hospital care as well as after the child's discharge from the PICU [14]. Factors identified as stressful for the parents include an unfamiliar high‐tech environment, which can be frightening for both the parent and child, and fear for the child's survival and future development [2, 13, 15]. The acute stress reaction can sometimes last up to several months or even years after the child's hospitalization and develop into post‐traumatic stress [15, 16]. The parents may have difficulties in comprehending the treatment and understanding information, as well as not having the courage or strength to participate in the child's care [17, 18]. Improving satisfaction with care has been shown to reduce parental stress [18] and one way to do this and thereby the quality of care is by working according to the principles of patient and family‐centred care (PFCC), where the child and the family are valued as equally important in the care team [19, 20]. The principles of PFCC are based on “*respect and dignity”*—professionals should respect and recognize the patient and family's viewpoint; “*information sharing”* exchange of information between professionals, patient and family; “*participation”* encouraging patient and families to take part in decisions about the care process as well as in care; *“collaboration”* partnership between professionals, patient and families in the development of policies and research as well as evaluation of care; and so on [20]. When those principles are practiced, professionals, patients, and families benefit as care is provided in partnership between all participants [21]. This approach can help reduce stress and increase satisfaction among the parents and the child being cared for [22].

To further develop PFCC in the clinical environment, we need to measure and understand which concepts of PFCC are realized in practice and which are lagging [20]. Parents play a significant role in their child's care, especially when the child is sedated and/or intubated or at a younger age. The parents represent both themselves and their child. Based on current knowledge, there is a lack of psychometrically tested Swedish questionnaires measuring parental satisfaction with FCC within the paediatric intensive care context. To our knowledge, only the EMPATHIC‐30 questionnaire has been identified as a reliable instrument for this purpose [23]. Based on EMPATHIC‐30, the purpose of this study was to explore parental satisfaction with paediatric intensive care.

## 2 Method

### 2.1 Design and Setting

For this cross‐sectional study, data were collected at two level III PICUs in Sweden. The two units have approximately 700 admissions in total per year. Children are admitted both from their catchment area as well as from other hospitals, and occasionally also other countries. Each unit has the capacity to admit 8–12 children, depending on the unit's current staffing. The PICUs have both single‐ and multi‐bedrooms. There are no visiting restrictions for family members. However, during the COVID‐19 pandemic in 2020, only one parent was allowed to stay with the child at a time. In the patient's room, there is an armchair available for parents to rest. Overnight accommodation is offered in the hospital area at the Ronald McDonald House located near the PICU. Parents are usually not invited to participate in daily ward rounds, but they are informed afterward by the physician or someone from the health care professional team. Parents are usually offered the opportunity to participate in the child's daily care, and they are also allowed to stay in the room when/if resuscitation is carried out. The health care professional team in this study is comprised of physicians, registered nurses, and assistant nurses.

### 2.2 Instrument

The Swedish version of the *Empowerment of Parents in the Intensive Care‐30* (EMPATHIC‐30) questionnaire was used in this study [24]. It is an instrument measuring parents' satisfaction with paediatric intensive care originally developed by Latour, Duivenvoorden [23]. It has been translated into several languages and is widely used in many countries [24, 25, 26]. The Swedish version was evaluated with acceptable psychometric properties [27].

EMPATHIC‐30 is self‐reported and based on the principles of family‐centred care. It includes 30 items divided into five domains: *information* (five items), *care and cure* (eight items), *organization* (five items), *parental participation* (six items), and *professional attitude* (six items). The items are answered using a 6‐graded Likert scale, ranging from one (certainly no) “strongly disagree” to six (certainly yes) “strongly agree”, a value of three is assigned to “neutral”. In cases where a response does not apply, the option “*not applicable”* (NA) is also available. According to the scoring method, a mean value is calculated for the total scale as well as for each domain. In addition to the EMPATHIC‐30 questionnaire, five questions are incorporated. Two questions pertain to the parents' inclination to recommend the ward to others and are responded to using a 6‐graded Likert scale. Furthermore, two questions concerning the overall experience of the nurses/assistant nurses and physicians were answered using a 10‐graded Likert scale, where the value one represents “very poor” and the value 10 represents “excellent”. Additionally, there are five open‐ended questions where parents can freely describe their experience during their child's hospitalization, as well as single items describing demographic data.

### 2.3 Sample

Both parents of children < 18 years of age hospitalized at the PICU for ≥ 48 h were individually invited to participate in the study. The inclusion criteria were that the parents should speak and understand Swedish. Exclusion criteria were parents whose child died during the care period at the PICU. In total, 234 questionnaires were distributed, and 100 were returned, corresponding to a 42.73% participation rate. However, three questionnaires were excluded because they did not fulfil the inclusion criteria (*n* = 2) or were returned with no answers (*n* = 1). In total, 97 questionnaires were included in the present study.

### 2.4 Data Collection

Data collection was carried out between February 2018 and September 2020. All parents who met the inclusion criteria were eligible for participation at the time of the child's discharge. A nurse or an assistant nurse informed the parents about the study and asked the parents if they were interested in receiving more information regarding the study. The interested parents received written information about the study, a consent form, the questionnaire, and a pre‐stamped envelope in which to return the questionnaire and the informed consent form. Parents who had not been asked at the PICU in connection with their child's discharge were invited to participate via a letter sent to their home address by the unit's secretary. The letter included the same information as mentioned above as well as additional written information that corresponded to the oral information that was given to the parents by the nurse/assistant nurse at the unit.

### 2.5 Data Analysis

The data were analysed using IBM SPSS Statistics Version 27 [28]. Demographic data were presented as frequency, percentage, mean and standard deviation, median, and min‐max. Comparisons between groups were analysed using the Mann–Whitney U‐test, as the data were not normally distributed. The significance level was set at *p* < 0.05.

## 3 Results

The results are based on 97 questionnaires representing parents of 55 children that were hospitalized at a PICU. Of the included parents (*n* = 97), 82 were parental couples (*n* = 41 couples). In total, 50 questionnaires were completed by mothers and 47 by fathers. Initial comparisons between mothers and fathers, as well as between parental couples, revealed no significant differences in satisfaction scores. Consequently, all responses were pooled for the main analysis.

### 3.1 Parents' and Children's Characteristics

There was an even distribution between mothers (*n* = 50) and fathers (*n* = 47), and their average age was comparable. The variation in age among the children was 3 days to 15 years, and 65% (*n* = 36) of the children were 6 months or younger. The median LOS was 7 days (interquartile range: 5.00–16.25 days). The 75th percentile for LOS was 16.25 days; this value was used as a cut‐off for identifying prolonged hospital stay, as this percentile reflects the upper quartile of LOS in the current cohort. Children with LOS longer than 16.25 days were classified as long‐stay patients (*n* = 14, 25%) of the total cohort (*n* = 55). Twelve children (22%) had previous experience of paediatric intensive care, and 23 (42%) had planned admission because of aftercare following major surgery. Parents' and children's characteristics are presented in Table 1.

**TABLE 1:** Parents' and children's characteristics.

Parents' characteristics *n* = 97 (*n*, %)	Mother *n* = 50 (51.5%)	Father *n* = 47 (48.5%)
Age years		
Mean, (SD)	36.9 (6.3)	37.7 (7.9)
Min‐max	23.0–52.0	25.0–61.0
Nationality, *n* (%)		
Swedish	44 (88.0)	41 (87.2)
European	6 (12.0)	4 (8.5)
Other	0 (0.0)	2 (4.3)
Education^a^, *n* (%)		
Elementary school	3 (6.1)	2 (4.3)
Upper secondary school	14 (28.6)	18 (39.1)
University	31 (63.3)	25 (54.3)
Other Education	1 (2.0)	1 (2.2)
Children's characteristics (*n*, %)	*n* = 55	%
Siblings in the family	41	75.5
Treated on ventilator	53	96.4
Prior experience of PICU care	12	21.8
Planned admission	23	41.8
Admission cause (*n*, %)	*n* = 55	%
Heart disease	34	61.8
Lung disease	2	3.6
Sepsis	1	1.8
Infection	6	10.9
Tumour	2	3.6
Undetermined	10	18.2
Children's length of stay (*n* = 55)	Days	
Min‐max	2.0–84.0	
Mean (SD)	11.9 (14.1)	
Median	7.0	

^a^ Internal missing mother *n* = 1; father *n* = 1.

### 3.2 Parents' Overall Satisfaction

The overall satisfaction with care was evaluated as high by the parents, both according to total scale (5.53), domains (5.42–5.77) (Table 2), as well as for single items (4.44–5.99) (Table 3). The domain *professional attitude* had the highest satisfaction score (5.77). *Care and cure* had the lowest satisfaction score among parents (5.42) closely followed by the domain *parental participation* (5.44) (Table 2). There was a low internal missing (*n* = 14) distributed over 12 items (item numbers 19–30, Table 3).

**TABLE 2:** Satisfaction scores total scale and domains.

EMPATHIC‐30	Mean	95% CI	SD	Median	Min‐Max
Total scale	**5.53**	**5.42–5.65**	**0.51**	**5.68**	**2.97–6.00**
Domains					
Information	5.46	5.28–5.59	0.67	5.60	2.20–6.00
Care and Cure	5.42	5.27–5.57	0.65	5.62	2.38–6.00
Organization	5.66	5.55–5.76	0.46	5.80	3.80–6.00
Parental participation	5.44	5.30–5.59	0.64	5.66	2.67–6.00
Professional attitude	5.77	5.68–5.87	0.42	6.00	3.50–6.00

**TABLE 3:** Satisfaction scores (given as mean and SD) of parents on item level (*n* = 97).

Items^a^	Mean	SD	Min‐Max	NA^b^
We felt welcome when we arrived at the clinic	5.67	0.86	1–6	0
2 The physicians clearly explained the effects of our child's treatment	5.54	0.73	3–6	0
3 We received clear information about examinations and treatments	5.57	0.80	2–6	0
4 We received clear information about the effects of the medicines	5.25	1.04	1–6	0
5 The collaboration between physicians and nurses was good	5.76	0.55	3–6	1
6 The health care team attempted to prevent and manage our child's pain	5.90	0.36	4–6	1
7 We were involved in the decision‐making process of our child's care and treatment	5.06	1.51	1–6	5
8 We were encouraged to stay with our child	5.73	0.70	2–6	0
9 During intensive procedures, we were able to stay and remain close to our child	5.55	0.97	1–6	3
10 The health care team worked in a hygienic way	5.90	0.33	4–6	0
11 The health care team ensured that our child's privacy was respected	5.75	0.64	2–6	0
12 The intensive care unit had a clean environment	5.90	0.30	1–6	0
13 The intensive care unit was easy to reach by phone	5.99	0.58	1–6	6
14 The sound level in the intensive care unit was reduced as much as possible	5.45	1.04	1–6	0
15 The space around our child's bed was adequate	5.19	1.14	1–6	0
16 The health care team worked efficiently	5.87	0.44	3–6	1
17 The health care team showed respect to our child and us	5.91	0.29	5–6	0
18 During the stay, the staff regularly asked us how we felt	5.00	1.33	1–6	2
19 We had daily conversations about our child's care and treatment with the physician	5.09	1.36	1–6	1
20 We had daily conversations about our child's care and treatment with the nurse/assistant nurse	5.86	0.55	3–6	1
21 Our child's well‐being was always taken into consideration by physicians	5.79	0.56	1–6	1
22 Our child's well‐being was always taken into consideration by nurse/assistant nurse	5.90	0.36	4–6	0
23 We knew every day who was responsible for our child by physicians	4.44	1.62	1–6	0
24 We knew every day who was responsible for our child by the nurse/assistant nurse	5.47	1.20	1–6	0
25 We felt trust in physicians	5.86	0.37	4–6	0
26 We felt trust in the nurse/assistant nurse	5.78	0.52	4–6	0
27 We were shown sympathy by physicians	5.68	0.74	1–6	0
28 We were shown sympathy by the nurse/assistant nurse	5.78	0.63	2–6	0
29 We were prepared in good time before our child's transfer/discharge by physicians	4.93	1.52	1–6	1
30 We were prepared in good time before our child's transfer/discharge by nurse/assistant nurse	5.19	1.28	1–6	1

^a^ Items translated from the Swedish version.

^b^ NA = not applicable.

### 3.3 Potential Factors Associated With Parents' Satisfaction

Although parents showed high satisfaction with paediatric intensive care, there were significant differences when comparing parents of children with planned and unplanned admissions (Table 4). Specifically, parents of children who underwent planned admissions reported a significantly higher level of satisfaction in terms of being encouraged to stay close to their child (*p* = 0.017). Additionally, when evaluating the item concerning the ability to remain close to their child during intensive procedures, parents of children with planned admissions expressed higher levels of satisfaction (*p* = 0.015).

**TABLE 4:** Potential indicators for parents' satisfaction, comparison of selected items by admission type, length of stay, and child age.

Item	Group 1	Mean (SD)	Group 2	Mean (SD)	Comparison*, ^a^ group 1 and 2
Admission type					
We were encouraged to stay with our child	Planned admission (*n* = 51)	5.88 (0.43)	Unplanned admission (*n* = 46)	5.57 (0.89)	0.017
During intensive procedures, we were able to remain and stay close to our child	Planned admission (*n* = 51)	5.78 (0.64)	Unplanned admission (*n* = 46)	5.28 (1.21)	0.015
Length of stay					
We received clear information about the effects of the medicines	LOS ≤ 7 days (*n* = 57)	5.44 (0.95)	LOS > 7 days (*n* = 40)	4.97 (1.12)	0.008
During intensive procedures, we were able to stay and remain close to our child	LOS ≤ 7 days (*n* = 57)	5.79 (0.62)	LOS > 7 days (*n* = 40)	5.20 (1.27)	0.015
The health care team ensured that our child's and our privacy was respected	LOS ≤ 7 days (*n* = 57)	5.89 (0.41)	LOS > 7 days (*n* = 40)	5.55 (0.85)	0.003
Child age					
We received clear information about the effects of the medicines	≤ 6 months (*n* = 61)	5.02 (1.04)	> 6 months (*n* = 36)	5.64 (0.93)	0.001
We had daily conversations about our child's care with physicians	≤ 6 months (*n* = 61)	4.77 (1.45)	> 6 months (*n* = 36)	5.66 (0.97)	0.001
We knew every day who was responsible for our child by physicians	≤ 6 months (*n* = 61)	4.11 (1.69)	> 6 months (*n* = 36)	5.00 (1.35)	0.018
We were prepared in good time before our child's transfer/discharge by physicians	≤ 6 months (*n* = 61)	4.70 (1.48)	> 6 months (*n* = 36)	5.31 (1.55)	0.016
We were prepared in good time before our child's transfer/discharge by the nurse/assistant nurse	≤ 6 months (*n* = 61)	4.97 (1.33)	> 6 months (*n* = 36)	5.57 (1.12)	0.016

* *p* < 0.05.

^a^ Mann–Whitney U‐test.

Parents of children with LOS < 7 days reported significantly higher satisfaction concerning privacy, the provision of information about medications, and the opportunity to stay close to their child during intensive procedures. Moreover, the age of the child emerged as a significant factor influencing parental satisfaction; parents of children older than 6 months demonstrated higher levels of satisfaction (Table 4).

### 3.4 Items With Lower Satisfaction Levels

Although parents demonstrated an overall high satisfaction with care, items with a lower satisfaction level (Likert scale 1–3) were revealed. Those items included involvement in decision‐making (Q7), communication about a parent's well‐being (Q18), communication regarding their child's care and treatment (Q19), information about the child's responsible physician (Q23), and preparation of the child's transfer/discharge from the PICU (Q29) (Table 3).

## 4 Discussion

The results demonstrated that parents had an overall high level of satisfaction with their child's paediatric intensive care with mean values ranging between 5.42 and5.77 for the domains and 5.53 for the total scale. The domains *professional attitudes* (5.77), *organization* (5.66) and *information* (5.46) had the highest values. A similar result has been shown in other studies. However, in those studies, the domain *organization* has not shown the correspondingly high value of satisfaction as in the present study. Differences in the countries' way of organizing health care services and paediatric intensive care may be an explanation for this difference [24, 29, 30].

The present study revealed that the child's age, LOS, and planned or unplanned admission were associated with the parent's satisfaction level. This corresponds with previous knowledge about factors that might affect parents' satisfaction with care [11, 31, 32]. Consistent with previous studies [33, 34], the present study revealed that parents with planned admissions reported higher satisfaction regarding their ability to be close to their child during the PICU stay and also being present during intensive procedures, compared to parents with unplanned admissions. An influencing fact for higher satisfaction reported by parents of children with planned admission may be that they were informed before the child's admission and therefore knew what to expect [33, 34]. When they were offered the chance to visit the unit before admission, it allowed them to see the hospital environment and prepare themselves. As a result, they were more informed and had a chance to ask questions compared to the parents with unplanned admission—this could be a factor that may have an impact on higher satisfaction levels [9, 33].

In addition, the present study revealed that parents with a child that had LOS less than or equal to 7 days were more satisfied both in terms of being present during intensive interventions and medical information as well as when their privacy was respected by the health care team [35]. Studies have confirmed that the stress level for parents when their child is being cared for in the PICU is high [12]; hence it is possible that longer LOS can affect the parents' stress and thereby their experience of satisfaction [36]. Another factor contributing to increased levels of stress may be the extended exposure to a high‐technological environment with stress‐inducing elements. In addition, witnessing their child's critical condition and the uncertainty about the child's survival are critical factors [15, 37]. Consistent with earlier research, our results showed that parents of children 6 months or younger reported lower satisfaction with medical information, communication, and discharge preparation [11]. The reason might be that parents of younger children often face greater uncertainty and anxiety [38], which can affect their satisfaction with the care provided. With this knowledge, interventions targeted towards parents with younger children might include planned daily communication between health care professionals and parents to identify support needs.

Person and Family‐Centred Care prioritizes the active involvement of patients and their families in decision‐making and care processes. It emphasizes respectful, responsive collaboration between health care professionals and families, ensuring that care aligns with the family's needs and preferences [20]. The EMPATHIC‐30 questionnaire is based on the principles of FCC designed to measure parental satisfaction in paediatric intensive care units. The results of EMPATHIC‐30 are thereby also an indicator of how FCC principles are realized in paediatric intensive care. Information sharing focuses on the “*exchange of information between professionals, patient and family”* [20] and this was revealed as one area for improvement in the present study. Furthermore, even though the average score difference was slight, all items were reviewed to identify possible areas needing improvements which rendered insight into important areas that could be improved in clinical practice to better meet parents' needs. This revealed that parents did not always know which doctor/nurse or assistant nurse was responsible for their child. Furthermore, they did not feel prepared for discharge. This can imply a lack of person‐centred communication [39, 40] which is a cornerstone of FCC [41]. Another area for improvement might be to further involve parents in decision‐making processes in line with the concept of *participation* in PFCC [20]. Parents in the present study were offered to participate in the child's daily care but they were not invited to participate in daily ward rounds where decisions about their child's care were taken. Family‐centred multidisciplinary rounds (FCR) could be one way to increase parents' participation in the decision‐making process. Earlier research has shown that FCR improves parental satisfaction and that families like to be a part of daily ward rounds [35, 42].

## 5 Limitations

Among the included parents, 82 were parental couples who may have influenced each other and thereby the results when answering the questionnaire. To reduce potential influence, parents were asked to complete the questionnaires separately. However, there might be a risk of influence, particularly given that parents in a shared situation may engage in discussions about their experience before answering the questionnaire. This is an inherent challenge in studies involving parental dyads [43]. Although their shared experiences might lead to similar answers, we believe that individual perceptions of care can still vary based on factors such as perceived gender roles, emotional processing, and involvement in the child's care [44, 45]. This assumption is supported by previous research, which was conducted in Swedish paediatric care, that revealed a significant difference between mothers' and fathers' satisfaction [41].

Furthermore, the inclusion period was long, which may have had an impact on the results, as both educational interventions in the staff group and working conditions at the PICUs may have occurred during that time. Likewise, it should be considered that during the data collection period, the COVID‐19 pandemic broke out. This implied that only one parent at a time was allowed to be present at the PICU. This lasted for 7 months of the data collection period and might have affected the parents' satisfaction and therefore the results of the study. However, given this limited timeframe and the relatively small proportion of the sample affected, it is difficult to draw robust conclusions regarding the influence of the pandemic on parental levels of satisfaction in our dataset. Although the results in the present study are in line with earlier studies [11, 31, 32, 35] it should be noted that the sample size was small. The statistical analysis was chosen based on the sample size. Another limitation is the lack of ethnic diversity among parents. Parents with a different ethnicity than Swedish might have other experiences regarding satisfaction with care. Parents of children who died during the care period were excluded because of ethical reasons. Future research should include those parents as their experiences are important because of their vulnerable situation.

## 6 Implications for Practice

Although the results showed high values of satisfaction with average values ranging between 5.42 and5.77, it was possible to identify areas for improvement related to PFCC. In particular, regarding effective communication, which is crucial in the PICUs [39]. The findings of this study emphasize the need for health care organizations to prioritize communication strategies that actively involve parents in decision‐making and participating in the child's care. Parents who feel adequately informed and involved in the decision‐making process are more likely to be satisfied with the care provided to their child [8].

## 7 Conclusion

The overall satisfaction with paediatric intensive care was evaluated as high by parents participating in the study. However, the results revealed areas for improvement related to the principles of family‐centred care, above all in the areas of *information sharing*, where open and transparent communication emerged as being important as well as *participation*. This includes preparing parents before their child's discharge, providing information about the responsible physician for the day, and involving parents in decisions about their child's care.

## Author Contributions

The present study was designed by K.T., J.W., U.J. and P.L. K.T. was primarily responsible for the data collection, data analysis and writing the manuscript. J.W., U.J. and P.L. participated in the data analysis and reviewed the manuscript. All authors read and approved the final manuscript.

## Ethics Statement

The study was approved by the Swedish Ethical Review Authority, regional board in Lund (Ref.no. 2018/547,2019–04602; Date of approval: 24 August 2019) and was conducted following the Helsinki Declaration of 2013 (22). All parents who participated in the study were provided with both written and oral information about voluntary participation, the right to withdraw at any time without further explanation. As well as information about confidentiality. In accordance with ethical protocols, the process of obtaining written informed consent was undertaken. The manuscript has not been previously submitted to any other journal, nor is it currently undergoing the process of evaluation by any other publication. All data were coded to protect confidentiality.

## Conflicts of Interest

The authors declare no conflicts of interest.

## Data Availability

The information disclosed in this article is not accessible to the public, as it is subject to ethical approval and compliance with the General Data Protection Rules (GDPR). The dataset includes sensitive details obtained from parents during the hospitalization of their critically ill children. Despite the anonymization of the data, it still contains information that could potentially lead to the identification of specific individuals. Participants in the study were assured that the data would exclusively be utilized by the researchers for the specified purpose and would not be disseminated. Inquiries regarding access to the dataset should be directed to Karina Terp at karina.terp@med.lu.se.

## References

[1] L. P. Nelson , S. E. Lachman , S. W. Li , and J. I. Gold , “The Effects of Family Functioning on the Development of Posttraumatic Stress in Children and Their Parents Following Admission to the PICU,” Pediatric Critical Care Medicine 20, no. 4 (2019): e208–e215. 30951005 10.1097/PCC.0000000000001894 PMC7518640

[2] I. Debelić , A. Mikolčić , J. Tihomirović , et al., “Stressful Experiences of Parents in the Paediatric Intensive Care Unit: Searching for the Most Intensive PICU Stressors,” International Journal of Environmental Research and Public Health 19, no. 18 (2022): 11450. 36141723 10.3390/ijerph191811450 PMC9517134

[3] C. C. Cintra , P. C. R. Garcia , S. Brandi , F. Crestani , A. R. D. Lessa , and M. L. D. R. Cunha , “Parents' Satisfaction With Care in Pediatric Intensive Care Units,” Revista Gaúcha de Enfermagem 43 (2022): e20210003. 35613234 10.1590/1983-1447.2022.20210003.en

[4] Socialstyrelsen , Barn som söker hälso‐ och sjukvård – Meddelandeblad, (Artikelnummer 2020‐12‐7117) (Socialstyrelsen, 2020), https://www.socialstyrelsen.se/kunskapsstod‐och‐regler/omraden/barn‐och‐unga/barn‐och‐unga‐i‐halso‐‐och‐sjukvarden/.

[5] Convention, U.N , United Nations Convention on the Rights of the Child (UNICEF, 1989).

[6] R. Crow , H. Gage , S. Hampson , et al., “The Measurement of Satisfaction With Healthcare: Implications for Practice From a Systematic Review of the Literature,” Health Technology Assessment 6, no. 32 (2002): 1–244. 10.3310/hta6320 12925269

[7] A. Abidova , P. A. da Silva , and S. Moreira , “Predictors of Patient Satisfaction and the Perceived Quality of Healthcare in an Emergency Department in Portugal,” Western Journal of Emergency Medicine 21, no. 2 (2020): 391–403. 31999247 10.5811/westjem.2019.9.44667 PMC7081842

[8] V. Matziou , B. Boutopoulou , A. Chrysostomou , E. Vlachioti , T. Mantziou , and K. Petsios , “Parents' Satisfaction Concerning Their Child's Hospital Care,” Japan Journal of Nursing Science 8, no. 2 (2011): 163–173. 22117580 10.1111/j.1742-7924.2010.00171.x

[9] S. Tsironi and G. Koulierakis , “Factors Affecting Parents' Satisfaction With Pediatric Wards,” Japan Journal of Nursing Science 16, no. 2 (2019): 212–220. 30474265 10.1111/jjns.12239

[10] S. Çimke and S. Mucuk , “Mothers' Participation in the Hospitalized Children's Care and their Satisfaction,” 2017.

[11] A. Kruszecka‐Krówka , G. Cepuch , A. Gniadek , E. Smoleń , K. Piskorz‐Ogórek , and A. Micek , “Selected Predictors of Parental Satisfaction With Child Nursing Care in Paediatric Wards in Poland—Cross‐Sectional Study,” PLoS One 16, no. 11 (2021): e0260504. 34797888 10.1371/journal.pone.0260504 PMC8604320

[12] J. Mortensen , B. O. Simonsen , S. B. Eriksen , P. Skovby , R. Dall , and A. Elklit , “Family‐Centred Care and Traumatic Symptoms in Parents of Children Admitted to PICU,” Scandinavian Journal of Caring Sciences 29, no. 3 (2015): 495–500. 25236928 10.1111/scs.12179

[13] L. P. Nelson and J. I. Gold , “Posttraumatic Stress Disorder in Children and Their Parents Following Admission to the Pediatric Intensive Care Unit: A Review,” Pediatric Critical Care Medicine 13, no. 3 (2012): 338–347. 21499173 10.1097/PCC.0b013e3182196a8f

[14] L. B. Ferreira , J. Oliveira , R. Gonçalves , T. Elias , S. Medeiros , and D. Mororó , “Nursing Care for the Families of Hospitalized Children and Adolescents,” 2018.

[15] K. Terp and A. Sjöström‐Strand , “Parents' Experiences and the Effect on the Family Two Years After Their Child Was Admitted to a PICU—An Interview Study,” Intensive & Critical Care Nursing 43 (2017): 143–148. 28663106 10.1016/j.iccn.2017.06.003

[16] H. Erçin‐Swearinger , T. Lindhorst , J. R. Curtis , H. Starks , and A. Z. Doorenbos , “Acute and Posttraumatic Stress in Family Members of Children With a Prolonged Stay in a PICU: Secondary Analysis of a Randomized Trial,” Pediatric Critical Care Medicine 23, no. 4 (2022): 306–314. 35190503 10.1097/PCC.0000000000002913 PMC9071176

[17] Z. Alzawad , F. M. Lewis , I. Kantrowitz‐Gordon , and A. J. Howells , “A Qualitative Study of Parents' Experiences in the Pediatric Intensive Care Unit: Riding a Roller Coaster,” Journal of Pediatric Nursing 51 (2020): 8–14. 31835065 10.1016/j.pedn.2019.11.015

[18] S. Tsironi and G. Koulierakis , “Factors Associated With Parents' Levels of Stress in Pediatric Wards,” Journal of Child Health Care 22, no. 2 (2018): 175–185. 29277106 10.1177/1367493517749327

[19] C. Hsu , M. F. Gray , L. Murray , et al., “Actions and Processes That Patients, Family Members, and Physicians Associate With Patient‐ and Family‐Centered Care,” BMC Family Practice 20, no. 1 (2019): 35. 30803446 10.1186/s12875-019-0918-7 PMC6388493

[20] IPFCC , “Institute for Patient‐ and Family‐Centrered Care,” 2017.

[21] W. Smith , “Concept Analysis of Family‐Centered Care of Hospitalized Pediatric Patients,” Journal of Pediatric Nursing 42 (2018): 57–64. 30219300 10.1016/j.pedn.2018.06.014

[22] M. Park , T. T. T. Giap , M. Lee , H. Jeong , M. Jeong , and Y. Go , “Patient‐ and Family‐Centered Care Interventions for Improving the Quality of Health Care: A Review of Systematic Reviews,” International Journal of Nursing Studies 87 (2018): 69–83. 30056169 10.1016/j.ijnurstu.2018.07.006

[23] J. M. Latour , H. J. Duivenvoorden , D. Tibboel , J. A. Hazelzet , and EMPATHIC Study Group , “The Shortened EMpowerment of PArents in THe Intensive Care 30 Questionnaire Adequately Measured Parent Satisfaction in Pediatric Intensive Care Units,” Journal of Clinical Epidemiology 66, no. 9 (2013): 1045–1050. 23790723 10.1016/j.jclinepi.2013.02.010

[24] F. J. Gill , S. Wilson , L. Aydon , G. D. Leslie , and J. M. Latour , “Empowering Parents of Australian Infants and Children in Hospital: Translation, Cultural Adaptation, and Validation of the EMpowerment of PArents in the Intensive Care‐30‐AUS Questionnaire,” Pediatric Critical Care Medicine 18 (2017): e506–e513. 28906423 10.1097/PCC.0000000000001309

[25] F. J. Pilar Orive , J. Basabe Lozano , A. López Zuñiga , Y. M. López Fernández , J. Escudero Argaluza , and J. M. Latour , “Spanish Translation and Validation of the EMPATHIC‐30 Questionnaire to Measure Parental Satisfaction in Intensive Care Units,” Anales De Pediatría (Barcelona) 89, no. 1 (2017): 50–57, 10.1016/j.anpede.2017.08.006. 29108859

[26] A. Girch , R. C. A. Rippe , J. M. Latour , et al., “The German EMPATHIC‐30 Questionnaire Showed Reliability and Convergent Validity for Use in an Intermediary/General Pediatric Cardiology Unit: A Psychometric Evaluation,” Frontiers in Cardiovascular Medicine 9 (2022): 901260. 35811695 10.3389/fcvm.2022.901260 PMC9262329

[27] K. Terp , U. Jakobsson , J. Weis , and P. Lundqvist , “The Swedish Version of EMPATHIC‐30 Translation and Initial Psychometric Evaluation,” Scandinavian Journal of Caring Sciences 37 (2023): 805–811. 36951241 10.1111/scs.13166

[28] IBM Corp , “*IBM SPSS Statistics for Windows (Version 25.0) [IBM SPSS Statistics]*,” 2017.

[29] D. B. Gomez , S. A. Vidal , and L. C. Lima , “Brazilian Adaptation and Validation of the Empowerment of Parents in the Intensive Care‐Neonatology (EMPATHIC‐N) Questionnaire,” Jornal de Pediatria 93, no. 2 (2017): 156–164. 27565641 10.1016/j.jped.2016.06.007

[30] F. J. Pilar Orive , J. Basabe Lozano , A. López Zuñiga , Y. M. López Fernández , J. Escudero Argaluza , and J. M. Latour , “Spanish Translation and Validation of the EMPATHIC‐30 Questionnaire to Measure Parental Satisfaction in Intensive Care Units,” Anales de Pediatría 89, no. 1 (2018): 50–57. 29108859 10.1016/j.anpedi.2017.08.004

[31] M. Abuqamar , D. H. Arabiat , and S. Holmes , “Parents' Perceived Satisfaction of Care, Communication and Environment of the Pediatric Intensive Care Units at a Tertiary Children's Hospital,” Journal of Pediatric Nursing 31, no. 3 (2016): e177–e184. 26803562 10.1016/j.pedn.2015.12.009

[32] A. Elbilgahy and S. Hashem , “Mothers' Satisfaction With Care Provided for Their Children in Pediatric Intensive Care Unit,” Middle East Journal of Nursing 13, no. 2 (2019): 17–28.

[33] J. M. Latour , J. B. van Goudoever , B. E. Schuurman , et al., “A Qualitative Study Exploring the Experiences of Parents of Children Admitted to Seven Dutch Pediatric Intensive Care Units,” Intensive Care Medicine 37, no. 2 (2011): 319–325. 21063674 10.1007/s00134-010-2074-3 PMC3028069

[34] C. Mol , A. Argent , and B. M. Morrov , “Parental Satisfaction With the Quality of Care in a South African Paediatric Intensive Care Unit,” Southern African Journal of Critical Care 34, no. 2 (2018): 50–56.

[35] K. Terp , J. Weis , and P. Lundqvist , “Parents' Views of Family‐Centered Care at a Pediatric Intensive Care Unit‐A Qualitative Study,” Frontiers in Pediatrics 9 (2021): 725040. 34513770 10.3389/fped.2021.725040 PMC8424181

[36] E. Segers , H. Ockhuijsen , P. Baarendse , I. van Eerden , and A. van den Hoogen , “The Impact of Family Centred Care Interventions in a Neonatal or Paediatric Intensive Care Unit on Parents' Satisfaction and Length of Stay: A Systematic Review,” Intensive & Critical Care Nursing 50 (2019): 63–70. 30249426 10.1016/j.iccn.2018.08.008

[37] C. Grandjean , P. Ullmann , M. Marston , M.‐C. Maitre , M.‐H. Perez , and A.‐S. Ramelet , “Sources of Stress, Family Functioning, and Needs of Families With a Chronic Critically Ill Child: A Qualitative Study,” Frontiers in Pediatrics 9 (2021): 740598. 34805041 10.3389/fped.2021.740598 PMC8600118

[38] A. F. Beato and P. J. Rosa , “Profiling Parent's Responses to Children's Anxiety: A Qualitative Study Combined With Multiple Correspondence and Cluster Analyses,” Journal of Child and Family Studies 33 (2024): 2870–2886.

[39] L. V. Marino , N. Collaḉo , S. Coyne , et al., “The Development of a Communication Tool to Aid Parent‐Centered Communication Between Parents and Healthcare Professionals: A Quality Improvement Project,” Healthcare (Basel) 11, no. 20 (2023): 2706. 37893780 10.3390/healthcare11202706 PMC10606263

[40] AAP , “Patient‐ and Family‐Centered Care and the Pediatrician's Role,” Pediatrics 129, no. 2 (2012): 394–404. 22291118 10.1542/peds.2011-3084

[41] G. Mikkelsen and K. Frederiksen , “Family‐Centred Care of Children in Hospital ‐ a Concept Analysis,” Journal of Advanced Nursing 67, no. 5 (2011): 1152–1162. 21272055 10.1111/j.1365-2648.2010.05574.x

[42] D. Z. Kuo , L. L. Sisterhen , T. E. Sigrest , J. M. Biazo , M. E. Aitken , and C. E. Smith , “Family Experiences and Pediatric Health Services Use Associated With Family‐Centered Rounds,” Pediatrics 130, no. 2 (2012): 299–305. 22778299 10.1542/peds.2011-2623 PMC3408683

[43] C. Hill , K. A. Knafl , S. Docherty , and S. J. Santacroce , “Parent Perceptions of the Impact of the Paediatric Intensive Care Environment on Delivery of Family‐Centred Care,” Intensive & Critical Care Nursing 50 (2019): 88–94. 30061085 10.1016/j.iccn.2018.07.007 PMC7159251

[44] D. Pelchat , H. Lefebvre , and M. Perreault , “Differences and Similarities Between Mothers' and Fathers' Experiences of Parenting a Child With a Disability,” Journal of Child Health Care 7, no. 4 (2003): 231–247. 14636429 10.1177/13674935030074001

[45] T. Leemann , E. Bergstraesser , E. Cignacco , and K. Zimmermann , “Differing Needs of Mothers and Fathers During Their Child's End‐Of‐Life Care: Secondary Analysis of the “Paediatric End‐Of‐Life Care Needs” (PELICAN) Study,” BMC Palliative Care 19, no. 1 (2020): 118. 32753031 10.1186/s12904-020-00621-1 PMC7405340

